# Do regulatory tools instigate measures to prevent work-related psychosocial and ergonomic risk factors? A process evaluation of a Labour inspection authority trial in the Norwegian home-care services

**DOI:** 10.1186/s13104-022-06244-4

**Published:** 2022-11-18

**Authors:** Håkon A. Johannessen, Stein Knardahl, Jan S. Emberland, Øivind Skare, Bjørnar Finnanger Garshol

**Affiliations:** 1 Department of Work Psychology and Physiology, National Institute of Occupational Health, Majorstuen, 5330, N-0034 Oslo, Norway; 2 National Institute of Occupational Health, Majorstuen, 5330, N-0034 Oslo, Norway; 3 Department of Occupational Medicine and Epidemiology, National Institute of Occupational Health, Majorstuen, 5330, N-0034 Oslo, Norway

**Keywords:** Cluster randomised controlled trial, Process evaluation, Occupational health and safety

## Abstract

**Objective:**

There is a research gap regarding the way managers and employee representatives respond to Labour Authority interventions targeting work-related psychosocial and ergonomic risk factors. The present study aimed to determine if (I) labour inspections and (II) guidance-through-workshops led by inspectors were perceived by the target audience as equally useful and educational; and to determine if utility and enhanced knowledge were associated with the implementation of measures to prevent work-related risk factors. Finally, it aimed to determine if the managers in the intervention groups to a greater extent than the controls reported implementing such measures.

**Results:**

Managers and employee representatives in both intervention groups reported a high level of perceived utility as well as a high level of enhanced knowledge. Both utility (p < 0.05) and enhanced knowledge (p < 0.05) were significantly associated with the implementation of, or plans to soon implement, measures to improve working conditions. When compared to controls, implemented measures, or plans to implement measures, were reported significantly more frequently by managers in the inspection group (p < 0.05).

*Trial Registration* ClinicalTrials.gov ID: NCT03855163 Registered on February 26, 2019.

**Supplementary Information:**

The online version contains supplementary material available at 10.1186/s13104-022-06244-4.

## Introduction

Psychosocial and ergonomic work factors contribute to the risk of developing musculoskeletal and mental disorders [1–5], which in turn are leading causes of reduced work ability and increased sick leave and disability pension [6–9]. Working conditions among home-care workers have been characterised by both high physical workloads and adverse psychosocial conditions [10–13], which could explain the high levels of sickness absence and disability retirement observed in the sector [14, 15].

Recent reviews suggest that labour inspections improve compliance with occupational safety and health (OSH) requirements and may reduce the incidence of occupational injuries [16–18]. However, there is a paucity of properly designed studies that address the effectiveness of regulatory tools in improving OSH management of psychosocial risks [19], and primary prevention of work-related musculoskeletal and mental disorders [16, 18–20].

The present study was carried out as a part of the cluster randomized controlled trial in Norwegian home-care services “Effectiveness of the Labour Inspection Authority’s Regulatory Tools for Work Environment and Employee Health” (EAVH project) [21]. The EAVH project hypothesises that inspection and guidance will increase compliance with OSH legislation and regulations, which in turn will lead to improved psychosocial and ergonomic working conditions and prevent employee ill health and sickness absence.

The present study aimed to evaluate the intervention implementation by testing three hypotheses: (I) Labour inspections and guidance workshops are perceived as equally useful (utility) and educational (enhanced knowledge); (II) Utility and enhanced knowledge are associated with intention to implement or having implemented changes to the work environment; (III) Participants in the labour inspection and guidance groups are more likely to report intention to implement or having implemented changes to the work environment than those in the control group.

## Methods

### Study design

We conducted a survey study to evaluate the implementation of the planned interventions in the EAVH project. These were (I) labour inspection, (II) guidance-through-workshop, and (III) an online risk-assessment tool [21]. Table 1 provides a summary of the planned interventions, see protocol for more information [21].

**Table 1:** – A summary of the intervention components in the EAVH-project

Intervention component	Brief description	Delivered by	Delivery frequency
Labour inspection	Labour inspections conducted by trained inspectors using a standardized checklist based on relevant laws and regulations	Inspectors from the Labour inspectorate authority	One inspection per participating organization/service with follow-up of any non-compliance
Guidance workshop	Half-day sessions were services present perceived issues related to psychosocial, organisational and mechanical factors at their workplace. Trained inspectors provided feedback on the issues presented based relevant laws and regulations and invited to discussion and reflection	Inspectors from the Labour Inspectorate Authority	Once per organization/service
Online risk assessment tool	Online risk-assessment tool comprising a customized checklist of psychosocial, organizational and mechanical risk factors in the home care services. Based on the answers supplied by the employers and employees the tool provides an action plan listing measures to reduce risks, the person responsible and a deadline for implementation	Web-based	Once per organization/service, but available throughout the intervention period
Control	Care as usual, i.e., no planned inspection etc	N/A	N/A

The clusters in the EAVH-project were municipalities, as they are the base administrative units of local government in Norway, and have a legal obligation to provide primary care, such as home care, for their inhabitants. Based on project sample size calculations, see protocol for details, 132 eligible municipalities and their home-care services were randomly allocated to four trial arms in September 2018 [21]. Additional file 1: Fig. S1 provides a flowchart of the trial. Because the Labour Inspection Authority needed time to plan the interventions, eligible municipalities were allocated before recruitment started. In November 2018, these municipalities were informed about the planned study and invited to participate.

Three months before the planned implementation of the interventions, 28 municipalities withdrew from the study. Based on the previous sample size calculations, we considered the remaining number of 104 municipalities as too low, and we therefore elected to drop the planned online risk-assessment tool intervention as this was entirely new and as such a less important tool for the Labour Inspectorate compared to labour inspections and guidance. To retain statistical power, the 23 municipalities in the online risk-assessment group were randomly reallocated to the other trial arms (Additional file 1: Fig. S1). This reallocation was conducted 2 months before recruitment of any home-care personnel and 4 months before implementation of the interventions. In the end, 96 of the 132 municipalities agreed to participate in the study, giving an overall response rate of 73%.

In March 2019, staff at the allocated home-care services were invited to participate. The Labour Inspection Authority subsequently conducted the inspections and guidance workshops after closure of the baseline survey (Additional file 2: Material 1), starting in May 2019. Three months after the interventions were implemented, we started to invite home-care services managers and employee representatives to answer the process evaluation questionnaire (Additional file 3: Material 2).

### Participants

Invited respondents of the present process evaluation were people from the participating services that had OSH-related roles, i.e., managers, employee representatives, or safety representatives, as systematic OSH management necessitates a cooperation between both employees and employers. In the labour inspection group, these were people who had interacted with, and provided information to, the inspectors. In the guidance-through-workshop group, these were people who had participated in the workshops. For the control group, we recruited managers in the services as we did not have any information available as to who were employee or safety representatives at those services.

In the labour inspection group, at least one respondent in 24 of the 30 municipalities completed the process evaluation questionnaire. Specifically, 39 (60%) of 65 managers, 14 (61%) of 23 employee representatives, 13 (50%) of 26 safety representatives, and 10 (28%) of 36 employees completed the questionnaire.

In the guidance-through-workshop group, at least one respondent in 28 of the 31 invited municipalities completed the process evaluation questionnaire. Specifically, 39 (63%) of 62 managers, 16 (50%) of 32 employee representatives, 21 (75%) of 28 safety representatives, and 17 (71%) of 27 employees completed the questionnaire.

In the control group, at least one respondent in 22 of the 35 invited municipalities completed the process evaluation questionnaire. In total, 52 (71%) of 73 managers completed the questionnaire.

### Process questionnaire measures

We elected to create our own assessment questions as we wanted to use questions tailored to this trial instead of more general standardised ones, and such questions could more adequately capture the project-specific context [22] Based on this process questionnaire (Additional file 3: Material 2), we created three dimensions, labelled utility, enhanced knowledge, and implemented measures. “Utility” aimed to capture whether the target audience perceived the interventions as useful and relevant in terms of OSH management at their workplace and was measured with five items (Table 2a). “Enhanced knowledge” aimed to capture whether the target audience perceived to have acquired improved skills to conduct OSH management at their workplace and was measured with three items (Table 2a). Response options for the items were 5-point scales ranging from (1) “a very small degree” to (5) “a very large degree”. Table 2a shows the item total correlations for these two constructs, that is, the correlation between an individual item and the total score without that item [22]. The correlations were high, indicating that the individual items are part of the same construct.

**Table 2:** .Individual item score and sum score for perceived utility and enhanced knowledge, and their associations with implementing preventive meassures

a—Individual item score and sum score for perceived utility and enhanced knowledge among managers and employee representatives							
Process evaluation items	Mean (SD)	Item total correlation	Mean (SD)	Item total correlation	MD (SE)	t-test	Sig
Utility (1-5)	**4.06 (0.51)**		**3.89 (0.71)**		**0.17 (0.10)**	**1.69**	**0.09**
At the time of the inspection/workshop, to what extent did you experience that							
The purpose of the inspection/workshop was disseminated in a clear and understandable way?	4.19 (0.56)	0.62	3.99 (0.76)	0.71			
The inspection/workshop addressed issues relevant for health and safety at your workplace?	4.37 (0.61)	0.58	4.07 (0.76)	0.81			
The health risk associated with the work environmental issues uncovered at your workplace were properly explained?	3.96 (0.67)	0.70	3.93 (0.74)	0.81			
The necessary actions needed to be taken to provide working conditions in line with OSH legislation and regulation was disseminated in a clear and understandable way?	3.92 (0.73)	0.69	3.83 (0.95)	0.82			
The inspection/workshop provided useful information for a systematic approach to health, environment, and safety management at your workplace?	3.95 (0.70)	0.60	3.67 (0.95)	0.77			
Enhanced knowledge (1–5)	**3.86 (0.65)**		**3.66 (0.86)**		**0.19 (0.13)**	**1.51**	**0.13**
Overall, has the inspection/workshop, contributed to							
Increased awareness of the importance of conducting work environmental risk assessments?	4.10 (0.68)	0.67	3.78 (0.94)	0.86			
Increased skills to improve your work environment?	3.73 (0.75)	0.77	3.60 (0.94)	0.84			
Enhanced knowledge of work environmental laws and regulations?	3.73 (0.79)	0.76	3.61 (0.91)	0.91			

b—Utility and enhanced knowledge as predictors of implemented measures, or such plans, among managers and employee representatives							
	N	Coefficient	S.E	Sig			
*Utility (1–5)*	141	0.64	0.32	0.04			
*Enhanced knowledge (1–5)*	142	0.82	0.27	0.01			

Bold values denotes the sum score

“Implemented measures” was assessed with a single item. The general wording was: “Have you recently implemented, or are you in the near future planning to implement, measures to improve the working environment at your workplace?” (Table 3A). For both intervention groups, the wording referred to the period after the intervention had been implemented. The response options were “yes”, “no” and “do not know”. To define the reference period for those in the control group, the following response options were possible: “yes, we have recently implemented measures”; “yes, we are in the process of implementing measures”; “yes, we are planning to implement measures by 2019”; “no”; “do not know”. The question was recoded into a dichotomous variable (yes/no). Those who responded “yes”, received follow-up questions pertaining to what kind of measures that either were or planned to be implemented (Table 3B–C).

**Table 3:** – Associations between intervention groups and self-report of implemented measures, or such plans, among home-care services managers

**A**		Have you implemented, or are you in near future planning to implement, measures to improve the working environment at your workplace?^a^					
		N	Yes n	%	Coefficient	S.E	Sig
Intervention groups		124	105	85			
Inspection		36	35	97	2.12	1.07	0.048
Guidance-through-workshops		36	28	78	−0.18	0.53	0.732
Controls		52	42	81	Reference		
Missing		6					

**B**		(If yes, what kind of measures) …identifying hazards and assessing the risks at your workplace?					
Intervention groups		105	66	63			
Inspection		35	27	77	1.31	0.51	0.010
Guidance-through-workshops		28	19	68	0.84	0.51	0.098
Controls		42	20	48	Reference		

**C**		(If yes, what kind of measures) …develop plans for a systematic approach to occupational safety and health management?					
Intervention groups		105	38	36			
Inspection		35	13	37	0.17	0.48	0.727
Guidance-through-workshops		28	11	39	4.07	0.51	0.611
Controls		42	14	33	Reference		

^a^Specified as post-intervention for the inspection and guidance-through-workshop groups

### Intervention implementations

Data on conducted inspections and breaches of OSH regulations were provided by the Labour Inspection Authority. The inspectors applied a standardised checklist which comprised items relevant to psychosocial and mechanical working conditions (Additional file 4: Table S1) to record compliance with OSH regulations within the municipal home-care services. The items represent compliance with a specific relevant legal requirement, and any non-compliance would trigger a formal order and have legal ramifications for the service enterprise.

Inspections were conducted as planned in 29 of the 30 municipalities. One municipality did not receive an inspection visit, as the municipality requested a postponement due to ongoing reorganization. Contraventions of OSH-requirements were detected in 28 of the 29 inspected municipalities (Additional file 4: Table S1). All these municipalities had at least one contravention of sufficient gravity to result in an order. The mean number of contraventions per municipality was 7 (standard deviation (SD) 4).

In the *guidance-through-workshop group*, two specific process questions were posed to managers and employee representatives: (1) “Did you prepare and hold a presentation on relevant issues arising from your own work environment” and (2) “Did two trained labour inspectors give guidance based on the issues presented at the workshop” (Additional file 3: Material 2). The intervention was conducted as intended in 26 of the 28 municipalities that completed the process questionnaire.

### Statistical analyses

All analyses were performed using IBM SPSS Statistics (version 25; IBM Corp., Armonk, NY, USA). Item-total correlations were computed to explore whether a specific item is measuring the same construct as the other items included [23]. Student’s *t* test was used to compare means. Associations between variables were calculated by logistic regression analyses.

## Results

Equal high levels of utility (mean difference (MD) 0.17, t = 1.69, p > 0.05) and enhanced knowledge (MD 0.19, t = 1.51, p > 0.05) were reported by managers and employee representatives in both intervention groups (Table 2a). Both utility (p < 0.05) and enhanced knowledge (p < 0.05) were significantly associated with self-report of implemented measures or plans for implementing measures. When compared to managers in the control group, managers in the inspection group reported significantly more frequently to have implemented, or having plans to soon implement, preventive measures (p < 0.05) (Table 3A). A corresponding finding was not present between managers in the guidance-through-workshop group and managers in the control group (p > 0.05). Regarding the nature of these preventive measures (Table 3B–C), significantly more managers in the inspection group confirmed to have implemented, or having plans to implement soon, measures of hazards identification and risk assessment (p < 0.05). A corresponding finding was not present between managers in the guidance-through-workshop group and managers in the control group (p > 0.05). There were no differences between the intervention groups and control group regarding developing plans for a systematic approach to OSH management (p > 0.05).

### Discussion

The way managers and employees with OSH responsibilities perceive and respond to labour inspectorate interventions targeting ergonomic and psychosocial risks at work is poorly understood [19, 24]. This study revealed that both managers and employee representatives in the home-care sector experienced the interventions provided by the Labour Inspection Authority as beneficial for managing workplace safety and health.

By law, it is essential and required, for enterprises to ensure a systematic, well-documented, and targeted approach to health, environmental, and safety activities at the workplace. For managers this includes an obligation to identify hazards and assess OSH risk factors. When compared to controls, significantly more managers from the inspection intervention group confirmed having implemented, or having plans to soon implement, preventive measures to ensure such an approach to OSH management (Table 3B). This finding may suggest that participating in the inspection intervention aided the managers to focus on relevant areas for change, whereas the lack of exposure to the intervention in the control group did not prompt such considerations.

There are few previous experimental or quasi-experimental studies that have addressed effectiveness of OSH management of psychosocial risk factors at work [19, 24]. The few studies that exist show results in line with findings in the present study [24–27], e.g., Weissbrodt and colleagues found that inspections improved OSH management, increased ability in psychosocial issues, perceived willingness to act, in addition to implementation of several psychosocial risk management measures [24].

## Limitations

Self-reported data on enhanced knowledge and intentions to implement preventive measures may have been inflated by social desirability or demand characteristics [28, 29]. A structured interview or an examination of the managers and the employee representatives are probably a more valid approach to capture whether the interventions increased knowledge and instigated measures to improve working conditions.

We cannot discount that merely participating in the research project may have primed the control group to focus on work factors and instigate processes to implement changes. Still, implemented measures, or plans to implement measures, were significantly more frequently reported by managers in the inspection group than in the control group.

The present study could have been strengthened by including two measurement points, i.e., measures pre- and post-intervention. Yet, the random assignment of services to intervention and control group, ensures no pre-intervention difference between the groups pertaining to OSH management, and we can rule out systematic differences between the groups pertaining to known and unknown confounding or prognostic factors.

## Supplementary Information

**Additional file 1: Figure S1**. Flow of Clusters (municipal home-care services) and Participants (home-care workers) Through the Trial.

**Additional file 2.** Work environment and health questionnaire.

**Additional file 3.** Process evaluation Questionnaire.

**Additional file 4: Table S1.** Contravened regulations in the inspection group.

## Data Availability

Data will be available 3 years after study completion. Data access request will be reviewed by NSD—Norwegian Centre for Research Data. URL: https://nsd.no/nsd/english/index.html.

## Abbreviations

OSH: Occupational safety and health
EAVH: Effectiveness of the labour inspection authority’s regulatory tools for work environment and employee health
M: Mean
SD: Standard deviation
MD: Mean difference
OR: Odds ratio
