# Supplementary figures and images for: Do regulatory tools instigate measures to prevent work-related psychosocial and ergonomic risk factors? A process evaluation of a Labour inspection authority trial in the Norwegian home-care services
Source: BMC Res Notes. 2022 Nov 18;15:349. doi: 10.1186/s13104-022-06244-4 (PMC9673432; doi:10.1186/s13104-022-06244-4)

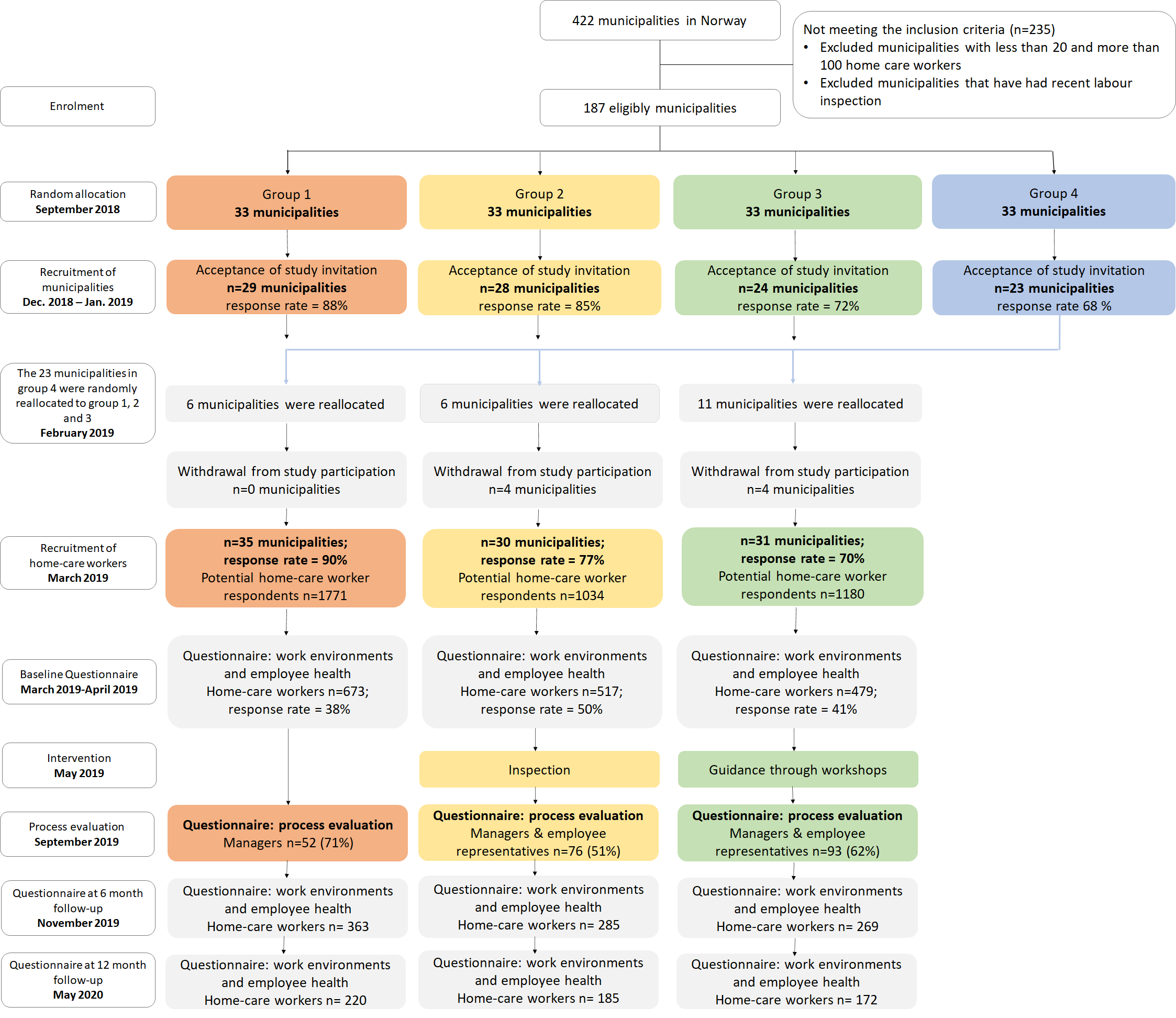

Supplement: Supplementary file 1 — Additional file 1: Figure S1. Flow of Clusters (municipal home-care services) and Participants (home-care workers) Through the Trial. [file 13104_2022_6244_MOESM1_ESM.tif]
