# Supplementary material for: Do regulatory tools instigate measures to prevent work-related psychosocial and ergonomic risk factors? A process evaluation of a Labour inspection authority trial in the Norwegian home-care services
Source: BMC Res Notes. 2022 Nov 18;15:349. doi: 10.1186/s13104-022-06244-4 (PMC9673432; doi:10.1186/s13104-022-06244-4)
Supplement: Supplementary file 2 — Additional file 2. Work environment and health questionnaire. [file 13104_2022_6244_MOESM2_ESM.docx]

Work environment and health

| **#1 Background** |
| --- |

**Year of birth**

| Use numbers (19xx) |
| --- |

|  |
| --- |

**Gender**

| Male |  |
| --- | --- |
| Female |  |

**Marital status**

| Unmarried |  |
| --- | --- |
| Married |  |
| Partnership |  |
| Widow/widower |  |
| Divorced |  |
| Separated |  |

**Formal education (number of years)**

| Elementary school (1-9 years) |  |
| --- | --- |
| High school (10- 12 years) |  |
| College or university, Bachelor’s Associate degree (13-16 years) |  |
| College or University, Master’s or Ph.D. (16 years or more) |  |

| **#2 Working hours** |
| --- |

How long have you been working for your current employer? (In whole years, if you have been working less than a year, please write 1)

|  | year |
| --- | --- |

What kind of employment contract do you have at your current workplace?

| Permanent employment |  |
| --- | --- |
| Temporary contract |  |
| Substitute/extra |  |
| Other |  |

**Do you have management responsibilities?**

| Do not have management responsibilities |  |
| --- | --- |
| Middle manager |  |
| Top manager |  |

**State the percentage of your employment (full time employment = 100%)?**

|  | % |
| --- | --- |

I do not have a fixed percentage of employment

What are your normal working hours?

| Daytime |  |
| --- | --- |
| Evening |  |
| Night-time |  |
| Shift work |  |
| No fixed working hours contract |  |

**If you work shifts, do you work:**

| Two-part shift (day and evening) |  |
| --- | --- |
| Three-part shift (day, evening, and night) |  |

**Have you worked at night in the past 12 weeks?**

| Yes |  |
| --- | --- |
| No |  |

**If yes, can you estimate how many nights you have worked in past 12 weeks?**

|  | nights |
| --- | --- |

**Can you influence decisions concerning your working hours/shift schedules?**

| Very seldom or never |  |
| --- | --- |
| Seldom |  |
| Sometimes |  |
| Often |  |
| Very often or always |  |

**Are you satisfied or dissatisfied with your current working hours/shift schedules?**

| Not at all satisfied |  |
| --- | --- |
| Partially satisfied |  |
| Satisfied |  |
| More than satisfied |  |
| Very satisfied |  |

| **#3 Job demands** |
| --- |

|  | Very seldom or never | Seldom | Sometimes | Often | Very often or always |
| --- | --- | --- | --- | --- | --- |
| Is your workload irregular so that the work piles up? | 1 | 2 | 3 | 4 | 5 |
| Do you have to work overtime? | 1 | 2 | 3 | 4 | 5 |
| Is it necessary to work at a rapid pace? | 1 | 2 | 3 | 4 | 5 |
| Do you have too much to do? | 1 | 2 | 3 | 4 | 5 |
| Does your work require quick decisions? | 1 | 2 | 3 | 4 | 5 |
| Does your work require maximum attention? | 1 | 2 | 3 | 4 | 5 |
| Does your work require complex decisions? | 1 | 2 | 3 | 4 | 5 |
| Are your work tasks too difficult for you? | 1 | 2 | 3 | 4 | 5 |
| Do you perform work tasks for which you need more training ? | 1 | 2 | 3 | 4 | 5 |
| Does your job require that you acquire new knowledge and new skills? | 1 | 2 | 3 | 4 | 5 |
| Is your work monotonous? | 1 | 2 | 3 | 4 | 5 |
| Do you have to repeat the same work procedure at intervals of a few minutes? | 1 | 2 | 3 | 4 | 5 |

| **#4 Mechanical working conditions** |
| --- |

| **How much of your time at work do you usually spend on:** | Never | Almost never | Around 1/4 of the time | Around half of the day | Around 3/4 of the time | Almost all of the time |
| --- | --- | --- | --- | --- | --- | --- |
| Travelling to clients? | 1 | 2 | 3 | 4 | 5 | 6 |
| Office work? | 1 | 2 | 3 | 4 | 5 | 6 |
| Clients/patients? | 1 | 2 | 3 | 4 | 5 | 6 |

| **In the course of your work…** | Never | Almost never | Around 1/4 of the time | Around half of the time | Around 3/4 of the time | Almost all of the time |
| --- | --- | --- | --- | --- | --- | --- |
| Do you need to squat or kneel? | 1 | 2 | 3 | 4 | 5 | 6 |
| Do you need to work standing up? | 1 | 2 | 3 | 4 | 5 | 6 |
| Do you work in positions where you are leaning forward without supporting yourself on your hands or arms? | 1 | 2 | 3 | 4 | 5 | 6 |

| **In an average working shift, how often do you have to:** | Never | Around 1-4 times | Around 5-9 times | Around 10-19 times | At least 20 times |
| --- | --- | --- | --- | --- | --- |
| Lift objects in awkward positions? | 1 | 2 | 3 | 4 | 5 |
| Lift anything that weighs more than 10 kg? | 1 | 2 | 3 | 4 | 5 |
| Manually transfer a client/patient between bed and chair? | 1 | 2 | 3 | 4 | 5 |
| Manually move a client/patient around on the bed, chair or wheelchair? | 1 | 2 | 3 | 4 | 5 |
| Do tasks that involve heavy physical efforts, without the option of using mechanical aids? | 1 | 2 | 3 | 4 | 5 |
| Do tasks that involve heavy physical efforts despite the availability of mechanical aids? | 1 | 2 | 3 | 4 | 5 |

**How would you rate your physical exertion during working with the clients/patients?**

| 0 | 1 | 2 | 3 | 4 | 5 | 6 | 7 | 8 | 9 | 10 |
| --- | --- | --- | --- | --- | --- | --- | --- | --- | --- | --- |
| Very, very light |  |  |  |  | Moderately strenuous |  |  |  |  | Very, very strenuous |

|  | To a small extent | To some extent | To a moderate extent | To a great extent | To a very great extent |
| --- | --- | --- | --- | --- | --- |
| To what extent is, the number of clients who require heavy physical efforts evenly distributed among the employees? | 1 | 2 | 3 | 4 | 5 |

| **#5 Emotion work** |
| --- |

|  | Very seldom or never | Seldom | Sometimes | Often | Very often or always |
| --- | --- | --- | --- | --- | --- |
| How often in your job do you have to suppress emotions in order to appear neutral on the “outside”? | 1 | 2 | 3 | 4 | 5 |
| How often in your job do you have to display emotions that do not agree with your actual feelings towards the clients? | 1 | 2 | 3 | 4 | 5 |
| How often in your job do you have to display pleasant emotions (i.e. friendliness) or unpleasant emotions (i.e. strictness) on the outside while actually feeling indifferent inside? | 1 | 2 | 3 | 4 | 5 |
| How often in your job do you have to display emotions that do not agree with your true feelings? | 1 | 2 | 3 | 4 | 5 |
| How often in your job do you need to deal with strong feelings such as sorrow, anger, desperation, frustration and so on from clients? | 1 | 2 | 3 | 4 | 5 |

| **#6 Role expectations** |
| --- |

|  | Very seldom or never | Seldom | Sometimes | Often | Very often or always |
| --- | --- | --- | --- | --- | --- |
| Have clear, planned goals and objectives been defined for your job? | 1 | 2 | 3 | 4 | 5 |
| Do you know what your responsibilities are? | 1 | 2 | 3 | 4 | 5 |
| Do you know exactly what is expected of you at work? | 1 | 2 | 3 | 4 | 5 |
| Do you have to do things that you feel should be done differently? | 1 | 2 | 3 | 4 | 5 |
| Are you given assignments without adequate resources to complete them? | 1 | 2 | 3 | 4 | 5 |
| Do you receive incompatible requests from two or more people? | 1 | 2 | 3 | 4 | 5 |

| **#7 Control at work** |
| --- |

|  | Very seldom or never | Seldom | Sometimes | Often | Very often or always |
| --- | --- | --- | --- | --- | --- |
| If there are alternative methods for doing your work, can you choose which method to use? | 1 | 2 | 3 | 4 | 5 |
| Can you influence the amount of work assigned to you? | 1 | 2 | 3 | 4 | 5 |
| Can you influence decisions concerning the persons you will need to collaborate with? | 1 | 2 | 3 | 4 | 5 |
| Can you decide when to be in contact with clients? | 1 | 2 | 3 | 4 | 5 |
| Can you influence decisions that are important for your work? | 1 | 2 | 3 | 4 | 5 |
| Can you set your own work pace? | 1 | 2 | 3 | 4 | 5 |
| Can you decide yourself when you are going to take a break? | 1 | 2 | 3 | 4 | 5 |
| Can you decide the length of your break? | 1 | 2 | 3 | 4 | 5 |
| Can you set your own working hours? | 1 | 2 | 3 | 4 | 5 |

| **#8 Predictability at work** |
| --- |

|  | Very seldom or never | Seldom | Sometimes | Often | Very often or always |
| --- | --- | --- | --- | --- | --- |
| Do you know in advance what kind of tasks to expect a month from now? | 1 | 2 | 3 | 4 | 5 |
| Do you know in advance who your co-workers will be a month from now? | 1 | 2 | 3 | 4 | 5 |
| Do you know in advance who your superior will be a month from now? | 1 | 2 | 3 | 4 | 5 |

| **#9 Positive challenges** |
| --- |

|  | Very seldom or never | Seldom | Sometimes | Often | Very often or always |
| --- | --- | --- | --- | --- | --- |
| Are your skills and knowledge useful in your work? | 1 | 2 | 3 | 4 | 5 |
| Is your work challenging in a positive way? | 1 | 2 | 3 | 4 | 5 |
| Do you consider your work meaningful? | 1 | 2 | 3 | 4 | 5 |

| **#10 New technology** |
| --- |

Over the past 12 months, have new technology or new administrative systems that affect you been implemented at your workplace?

| Yes |  |
| --- | --- |
| No |  |
| Not sure |  |

If new technology has been introduced, have you received the necessary training on how to use the new technology?

| Yes, very good |  |
| --- | --- |
| Yes, good |  |
| Moderate |  |
| No, bad |  |
| No, very bad |  |
| Not sure/do not know |  |

**If new technology has been introduced, have the employees (or employee representatives) been involved in the introduction of this new technology?**

| Yes |  |
| --- | --- |
| No |  |
| Not sure |  |

| **#11 Affiliation** |
| --- |

**How often do you consider leaving you current position?**

| Never |  |
| --- | --- |
| Rarely |  |
| Once in a while |  |
| Often |  |

**Are you currently applying for a job at a different employer, or do you expect to do so over the next couple of years?**

| No |  |
| --- | --- |
| Yes, currently applying |  |
| Yes, expect to apply |  |

| **#12 Social support** |
| --- |

|  | Very seldom or never | Seldom | Sometimes | Often | Very often or always |
| --- | --- | --- | --- | --- | --- |
| If needed, can you get support and help with your work from your co-workers? | 1 | 2 | 3 | 4 | 5 |
| If needed, are your co-workers willing to listen to your work-related problems? | 1 | 2 | 3 | 4 | 5 |

|  | Very seldom or never | Seldom | Sometimes | Often | Very often or always |
| --- | --- | --- | --- | --- | --- |
| Does your immediate superior distribute the work fairly and impartially? | 1 | 2 | 3 | 4 | 5 |
| Does your immediate superior treat the workers fairly and equally? | 1 | 2 | 3 | 4 | 5 |
| Is the relationship between you and your immediate superior a source of stress for you? | 1 | 2 | 3 | 4 | 5 |
| Does your immediate superior encourage you to participate in important decisions? | 1 | 2 | 3 | 4 | 5 |
| Does your immediate superior encourage you to speak up, when you have differing opinions? | 1 | 2 | 3 | 4 | 5 |
| Does your immediate superior help you develop your skills? | 1 | 2 | 3 | 4 | 5 |
| If needed, can you get support and help with your work from your immediate superior? | 1 | 2 | 3 | 4 | 5 |
| If needed, is your immediate superior willing to listen to your work-related problems? | 1 | 2 | 3 | 4 | 5 |
| Are your work achievements appreciated by your immediate superior? | 1 | 2 | 3 | 4 | 5 |

| **#13 Leadership** |
| --- |

| **Evaluate the following statements about your immediate superior’s management style.** | Very seldom or never | Seldom | Sometimes | Often | Very often or always |
| --- | --- | --- | --- | --- | --- |
| They get involved when things come up which are important to me or my work. | 1 | 2 | 3 | 4 | 5 |
| They are there when I need them. | 1 | 2 | 3 | 4 | 5 |
| They make decisions that have a large impact on my work. | 1 | 2 | 3 | 4 | 5 |
| They answer questions which are important to have a timely answer to. | 1 | 2 | 3 | 4 | 5 |

| **#14 Organizational climate** |
| --- |

|  | Very seldom or never | Seldom | Sometimes | Often | Very often or always |
| --- | --- | --- | --- | --- | --- |
| Have you noticed any inequalities in how men and women are treated at your workplace? | 1 | 2 | 3 | 4 | 5 |
| Have you noticed any inequalities in how older and younger employees are treated at your workplace? | 1 | 2 | 3 | 4 | 5 |
| At your organization are you rewarded (money, encouragement) for a job well done? | 1 | 2 | 3 | 4 | 5 |
| Are workers well taken care of in your organization? | 1 | 2 | 3 | 4 | 5 |
| To what extent is the management of your organization interested in the health and well-being of the personnel? | 1 | 2 | 3 | 4 | 5 |

| **#15 Organizational changes** |
| --- |

**Have there been any reorganizations at your workplace over the past three years that have affected your work situation?**

| Yes, at my work unit |  |
| --- | --- |
| Yes, at another work unit |  |
| No |  |

**If yes, to what degree have the reorganizations had impact on your work situation?**

| Very big impact |  |
| --- | --- |
| Big impact |  |
| Small impact |  |
| Very small impact |  |
| No impact |  |

**Has your employer implemented a process by which all those who are affected by the changes can be heard?**

| Yes |  |
| --- | --- |
| No |  |
| Not sure |  |

| **#16 Health** |
| --- |

How would you rate your health?

| Very good |  |
| --- | --- |
| Good |  |
| Moderate |  |
| Bad |  |
| Very bad |  |

| **Have you had any of the symptoms or complaints during the last 4 weeks?** | Not troubled | A little troubled | Moderately troubled | Seriously troubled | **If you have been troubled,** did the complaint get worse at work? |
| --- | --- | --- | --- | --- | --- |
| Headache | 1 | 2 | 3 | 4 | Yes No |
| Pain in the neck | 1 | 2 | 3 | 4 | Yes No |
| Pain in the shoulder, upper arm | 1 | 2 | 3 | 4 | Yes No |
| Pain in forarm, hand | 1 | 2 | 3 | 4 | Yes No |
| Back pain | 1 | 2 | 3 | 4 | Yes No |
| Pain in hips, legs, knees or feet | 1 | 2 | 3 | 4 | Yes No |

**Please circle the alternative that best describes** **your general pain intensity throughout the last week (0 means no pain and 10 means the worst possible pain).**

| 0 | 1 | 2 | 3 | 4 | 5 | 6 | 7 |  | 8 | 9 | 10 |
| --- | --- | --- | --- | --- | --- | --- | --- | --- | --- | --- | --- |
| No pain |  |  |  |  |  |  |  |  |  |  | Worst possible pain |

| **Below is a list of various problems. Have you suffered from any of the following during the last week?** | Not bothered | A little bothered | Moderately bothered | Seriously bothered | **If you have been troubled,** did the complaint get worse at work? |
| --- | --- | --- | --- | --- | --- |
| Nervousness or disquiet | 1 | 2 | 3 | 4 | Yes No |
| Consistently scared or anxious | 1 | 2 | 3 | 4 | Yes No |
| Feeling hopeless about the future | 1 | 2 | 3 | 4 | Yes No |
| Feeling down or heavy-hearted | 1 | 2 | 3 | 4 | Yes No |
| Often worried or upset | 1 | 2 | 3 | 4 | Yes No |

| **How often…** | Never/almost never | A couple of times a month | One or two times a week | Three or four times a week | Almost every day | **If you have been troubled,** did the complaint get worse at work? |
| --- | --- | --- | --- | --- | --- | --- |
| do you feel tired? | 1 | 2 | 3 | 4 | 5 | Yes No |
| are you physically exhausted? | 1 | 2 | 3 | 4 | 5 | Yes No |
| are you emotionally exhausted? | 1 | 2 | 3 | 4 | 5 | Yes No |
| do you think: "I can't take it anymore"? | 1 | 2 | 3 | 4 | 5 | Yes No |
| do you feel worn out? | 1 | 2 | 3 | 4 | 5 | Yes No |
| How often do you feel weak and susceptible to illness? | 1 | 2 | 3 | 4 | 5 | Yes No |

| **#17 Sleep** |
| --- |

**The questionnaire below contains six questions relating to sleep and tiredness. Please circle the alternative (number of days per week) that suits you best. 0 means no days during the course of a week, 7 means every day during the course of a week.**

| During the past month, how many days a week has it taken you more than 30 minutes to fall asleep after the light was switched off? | 1 | 2 | 3 | 4 | 5 | 6 | 7 |
| --- | --- | --- | --- | --- | --- | --- | --- |
| During the past month, how many days a week have you been awake for more than 30 minutes between periods of sleep? | 1 | 2 | 3 | 4 | 5 | 6 | 7 |
| During the past month, how many days a week have you woken up more than 30 minutes earlier than you wished to without managing to fall asleep again? | 1 | 2 | 3 | 4 | 5 | 6 | 7 |
| During the past month, how many days a week have you felt that you have not had enough rest after waking up? | 1 | 2 | 3 | 4 | 5 | 6 | 7 |
| During the past month , how many days a week have you been so sleepy/tired that it has affected you at school/work or in your private life? | 1 | 2 | 3 | 4 | 5 | 6 | 7 |
| During the past month, how many days a week have you been dissatisfied with your sleep? | 1 | 2 | 3 | 4 | 5 | 6 | 7 |

| **#18 Work ability** |
| --- |

**Assume that your work ability at its best has a value of 10 points. How many points would you give your current work ability? (0 means that you cannot currently work at all)**

| 0 | 1 | 2 | 3 | 4 | 5 | 6 | 7 | 8 | 9 | 10 |
| --- | --- | --- | --- | --- | --- | --- | --- | --- | --- | --- |
| Cannot currently work at all |  |  |  |  |  |  |  |  |  | Work ability at its best |

| **#19 Accidents** |
| --- |

**During the past 12 months, have you been injured as the result of an accident at work and which led to sick leave after the day of the accident?**

| Yes |  |
| --- | --- |
| No |  |

| **#21 Bullying and harassment** |
| --- |

**Bullying (harassment, psychological violence, badgering, offending somebody) is a problem at some work-places and for some workers. For something to be considered bullying, the offensive behaviour has to occur repeatedly over a period of time, and the person confronted has to experience difficulties defending himself/herself.**

**Have you been subjected to bullying or harassment from co-workers at your workplace during the last six months?**

| No |  |
| --- | --- |
| Yes, rarely |  |
| Yes, occasionally |  |
| Yes, several times a week |  |
| Yes, daily |  |

**Have you been subjected to bullying or harassment from a superior at your workplace during the last six months?**

| No |  |
| --- | --- |
| Yes, rarely |  |
| Yes, occasionally |  |
| Yes, several times a week |  |
| Yes, daily |  |

**If you have been subjected to bullying or harassment at the workplace, have you raised your concerns to someone who you think could stop the situation?**

| Yes, to my nearest supervisor |  |
| --- | --- |
| Yes, to the employee representative |  |
| Yes, to a colleague |  |
| Yes, to the occupational health care |  |
| No |  |
| Not sure/don’t remember |  |

**If concerns were raised, what happened with the issue the concerns were raised about?**

| It hasn’t happened again after I raised my concerns |  |
| --- | --- |
| The issue has diminished |  |
| Nothing has changed, I still experience bullying or harassment |  |
| The issue has worsened |  |
| Don’t know, not enough time has passed |  |

| **#22 Threats and violence** |
| --- |

**Over the past 12 months, have you been the victim of violence at the workplace?**

| No |  |
| --- | --- |
| Yes, it has happened once |  |
| Yes, once or several times a month |  |
| Yes, several times a week |  |

**If the answer is yes,** **who perpetuated you to violence?** (several answers are possible)

| A superior |  |
| --- | --- |
| A co-worker |  |
| A client or someone not employed at the workplace |  |

**Over the past 12 months, have you been threatened at the workplace in such a way that you felt scared?**

| No |  |
| --- | --- |
| Yes, it has happened once |  |
| Yes, once or several times a month |  |
| Yes, several times a week |  |

**If the answer is yes,** **who threatened you?** (several answers are possible)

| A superior |  |
| --- | --- |
| A co-worker |  |
| A client or someone not employed at the workplace |  |

**If you have experienced treats or violence at the workplace, have you raised your concerns to someone who you think could stop the situation?**

| Yes, to my nearest supervisor |  |
| --- | --- |
| Yes, to the employee representative |  |
| Yes, to a colleague |  |
| Yes, to the occupational health care |  |
| No |  |
| Not sure/don’t remember |  |

**If concerns were raised, what happened with the issue the concerns were raised about?**

| It hasn’t happened again after I raised my concerns |  |
| --- | --- |
| The issue has diminished |  |
| Nothing has changed, I still experience treats or violence |  |
| The issue has worsened |  |
| Don’t know, not enough time has passed |  |

|  |  |
| --- | --- |

| **#23 Unwanted sexual attention** |
| --- |

**Do you sometimes receive unwanted sexual attention, comments, etc. at your workplace?**

| No |  |
| --- | --- |
| Yes, it has happened once |  |
| Yes, once or more a month |  |
| Yes, once or more a week |  |

**If the answer is yes,** **who perpetuated the unwanted sexual attention?** (several answers are possible)

| A superior |  |
| --- | --- |
| A co-worker |  |
| A client or someone not employed at the workplace |  |

**If you have received unwanted sexual attention at work, have you raised your concerns to someone who you think could stop the situation?**

| Yes, to my nearest supervisor |  |
| --- | --- |
| Yes, to the employee representative |  |
| Yes, to a colleague |  |
| Yes, to the occupational health care |  |
| No |  |
| Not sure/don’t remember |  |

**If concerns were raised, what happened with the issue the concerns were raised about?**

| It hasn’t happened again after I raised my concerns |  |
| --- | --- |
| The issue has diminished |  |
| Nothing has changed, I still receive unwanted sexual attention |  |
| The issue has worsened |  |
| Don’t know, not enough time has passed |  |

| **#24 HSE** |
| --- |

**Over the past 12 month, have you had an appraisal interview?**

| Yes |  |
| --- | --- |
| No |  |
| Not sure |  |

**Over the past 12 month, have you participated in any kind of workplace interventions aiming to improve the work environment**

| Yes |  |
| --- | --- |
| No |  |
| Not sure |  |

**Does your company have a safety representative, i.e. someone who raises issues relating to the working environment on behalf of employees?**

| Yes |  |
| --- | --- |
| No |  |
| Not sure |  |

**Does your company have a working environment committee or other committees that deal with issues relating to the working environment?**

| Yes |  |
| --- | --- |
| No |  |
| Not sure |  |

| **#25 Raising concerns** |
| --- |

**Have you, in the past 12 months, discovered, experienced, or witnessed unacceptable conditions at your workplace which should have been stopped?**

Examples include breaches of rules and regulations regarding safety or the work environment, bullying, harassment, patient safety issues

| Yes |  |
| --- | --- |
| No |  |

**If yes, did you raise your concerns to someone who you think could stop the situation?**

| Yes |  |
| --- | --- |
| No |  |

**If concerns were raised, what happened with the issue the concerns were raised about?**

| It stopped |  |
| --- | --- |
| It got better |  |
| No change |  |
| It got worse |  |
| Don’t know, not enough time has passed |  |
