# Supplementary material for: Do regulatory tools instigate measures to prevent work-related psychosocial and ergonomic risk factors? A process evaluation of a Labour inspection authority trial in the Norwegian home-care services
Source: BMC Res Notes. 2022 Nov 18;15:349. doi: 10.1186/s13104-022-06244-4 (PMC9673432; doi:10.1186/s13104-022-06244-4)
Supplement: Supplementary file 3 — Additional file 3. Process evaluation Questionnaire. [file 13104_2022_6244_MOESM3_ESM.docx]

**Process evaluation questionnaires used in “Do regulatory tools instigate measures to prevent work-related musculoskeletal pain and mental distress? A process evaluation of a Labour Inspection Authority Trial in the home-care services”**

1. Questionnaire on the labour inspection conducted by the Labour Inspection Authority
   - Completed by participants from the Inspection-group
2. Questionnaire on the guidance workshop hosted by the Labour Inspection Authority
   - Completed by participants from the Guidance-through-workshop group
3. Questionnaire on systematic occupational safety and health management
   - Completed by participants from the Control-group

**Questionnaire on the labour inspection conducted by the Labour Inspection Authority**

**#1 What is your role in the occupational safety and health management at your workplace?** (Check all that apply)

| Manager (Unit) |  |
| --- | --- |
| Manager/Manager (Section) |  |
| Safety representative |  |
| Union representative |  |
| Other, please specify: |  |
| ………………………………………………………………………………………… |  |

**#2**

| ***At the time of the inspection, to what extent did you experience that…*** | To a very small degree | To a small degree | Neither | To a large degree | To a very large degrre |
| --- | --- | --- | --- | --- | --- |
| … the purpose of the inspection was disseminated in a clear and understandable way? | 1 | 2 | 3 | 4 | 5 |
| … the inspection addressed issues relevant for health and safety at your workplace? | 1 | 2 | 3 | 4 | 5 |
| … the health risk associated with the work environmental issues uncovered at your workplace were properly explained? | 1 | 2 | 3 | 4 | 5 |
| … the necessary actions needed to be taken to provide working conditions in line with occupation safety and health legislation and regulation was disseminated in a clear and understandable way? | 1 | 2 | 3 | 4 | 5 |
| … the inspection provided useful information for a systematic approach to health, environment, and safety management at your workplace? | 1 | 2 | 3 | 4 | 5 |
| …. the inspection was time consuming? | 1 | 2 | 3 | 4 | 5 |

**#3 What were the results of the labour inspection conducted at your workplace?**

| Formal order or warning of potential formal order  (Required to amend one or more breaches of regulation) |  |
| --- | --- |
| No formal orders, only oral/written information regarding the work environment |  |
| The results are not known at present |  |

**#4 How many work hours would you estimate you spent on the labour inspection?**

*This includes hours used from you received notice of the inspection and until the inspection visit was completed.*

|  | **Hours** |
| --- | --- |

**#5 Have you recently implemented, or are you in the near future planning to implement, measures to improve the working environment at your workplace after the labour inspection?**

| Yes |  |
| --- | --- |
| No |  |
| Do not know |  |

**#5a If yes, which measures have you implemented or are planning to implement?** (Check all that apply)

| Reinforce the work of safety representatives and/or the occupational health services |  |
| --- | --- |
| Identifying hazards and assessing the risks at your workplace |  |
| Develop plans for a systematic approach to occupational safety and health management |  |
| Conduct training/courses to enhance employee competencies |  |
| Make changes to the shift/work plan and/or the distribution of shifts |  |
| Make changes to the client list to ensure a more even distribution of clients among staff |  |
| Reorganise work tasks to reduce emotional load for employees |  |
| Clarify employees’ roles and responsibilities |  |
| Facilitate work tasks to avoid heavy lifting and/or monotonous work |  |
| Increase employee participation through strengthening the work environment committee |  |
| Develop plans and routines to protect employees from threats and violence |  |
| Develop plans and routines to protect employees from unwanted sexual attention |  |
| Other, please specify: | |

**#5b Where any of the measures mentioned above implemented before the labour inspection?**

| Yes |  |
| --- | --- |
| No |  |
| Do not know |  |

**#5c If you are not implementing any measures to improve the work environment, what is the reason behind this?**

| We recently implemented measures |  |
| --- | --- |
| We do not see the need to implement measures to improve the work environment |  |
| We had to postpone any implementation due to other resource demanding projects |  |
| Other, please specify:  ……………………….………………………………… | |

**#6 Have you recently sought information on occupational safety and health management?** (Check all that apply)

| No |  |
| --- | --- |
| Yes, from the occupational health service |  |
| Yes, from the municipality |  |
| Yes, from the Labour Inspection Authority web site |  |
| Yes, from the National Institute of Occupational Health website |  |
| Yes, from the “Idébanken” website |  |
| Other, please specify:  ……………………………………………… | |

**#7**

| **Overall, has the labour inspection contributed to…** | To a very small degree | To a small degree | Neither | To a large degree | To a very large degree |
| --- | --- | --- | --- | --- | --- |
| …increased awareness of the importance of conducting work environmental risk assessments? | 1 | 2 | 3 | 4 | 5 |
| … increased skills to improve your work environment? | 1 | 2 | 3 | 4 | 5 |
| …. enhanced knowledge of work environmental laws and regulations? | 1 | 2 | 3 | 4 | 5 |

**Questionnaire on the guidance workshop hosted by the Labour Inspection Authority**

**#1 What is your role in the occupational safety and health management at your workplace?** (Check all that apply)

| Manager (Unit) |  |
| --- | --- |
| Manager/Manager (Section) |  |
| Safety representative |  |
| Union representative |  |
| Other, please specify: |  |
| ………………………………………………………………………………………… |  |

**#2 Have representatives from your workplace recently attended a workshop on work environment hosted by the Labour Inspection Authority?**

| Yes |  |
| --- | --- |
| No |  |
| Do not know |  |

**#2a If yes, who attended the workshop on behalf of your workplace?** (Check all that apply)

| Manager (Unit) |  |  |  |
| --- | --- | --- | --- |
| Manager/Manager (Section) |  |  |  |
| Safety representative |  |  |  |
| Union representative |  |  |  |
| Occupational health service representative |  |  |  |
| Other, please specify: | | |  |
| ………………………………………………………………………………………… | | |  |

**#2b If you did not attend, what were the reasons for this?** (Check all that apply)

| It was not possible due to employee absence |  |  |  |
| --- | --- | --- | --- |
| The workshop was far away |  |  |  |
| We did not have an opportunity to prepare for the workshop |  |  |  |
| We did not have the resources (time, funds, etc) to attend |  |  |  |
| The workshop was not perceived as relevant for us |  |  |  |
| We did not receive sufficient information on from the Labour Inspection Authority |  |  |  |
| The time between being invited and the workshop itself was too short |  |  |  |
| Other, please specify: | | |  |
| ………………………………………………………………………………………… | | |  |

**Questions for those who attended the workshop hosted by the Labour Inspection Authority:**

**#3 When you participated in the workshop, did your workplace give a presentation of relevant issues from your own work environment?**

| Yes |  |
| --- | --- |
| No |  |
| Do not know |  |

**#3a If yes, did you receive any guidance from the Labour Inspection Authority on the basis of the presentation?**

| Yes, to a large degree |  |
| --- | --- |
| Yes, to some degree |  |
| Yes, to a small degree |  |
| No, we did not receive any guidance |  |

**#4**

| ***At the time of the workshop, to what extent did you experience that…*** | To a very small degree | To a small degree | Neither | To a large degree | To a very large degrre |
| --- | --- | --- | --- | --- | --- |
| … the purpose of the workshop was disseminated in a clear and understandable way? | 1 | 2 | 3 | 4 | 5 |
| … the workshop addressed issues relevant for health and safety at your workplace? | 1 | 2 | 3 | 4 | 5 |
| …the health risk associated with the work environmental issues presented from your workplace were properly explained? | 1 | 2 | 3 | 4 | 5 |
| …the necessary actions needed to be taken to provide working conditions in line with occupational safety and health legislation and regulation was disseminated in a clear and understandable way? | 1 | 2 | 3 | 4 | 5 |
| …the workshop provided useful information for a systematic approach to health, environment, and safety management at your workplace? | 1 | 2 | 3 | 4 | 5 |
| …participating in the workshop was time consuming? | 1 | 2 | 3 | 4 | 5 |

**#5 How many work hours would you estimate you spent on the workshop?**

*This includes hours used from you received the invitation until the workshop was completed.*

|  | **Hours** |
| --- | --- |

**#6 Have you recently implemented, or are you in the near future planning to implement, measures to improve the working environment at your workplace after attending the workshop?**

| Yes |  |
| --- | --- |
| No |  |
| Do not know |  |

**#6a If yes, which measures have you implemented or are planning to implement?** (Check all that apply)

| Reinforce the work of safety representatives and/or the occupational health services |  |
| --- | --- |
| Identifying hazards and assessing the risks at your workplace |  |
| Develop plans for a systematic approach to occupational safety and health management |  |
| Conduct training/courses to enhance employee competencies |  |
| Make changes to the shift/work plan and/or the distribution of shifts |  |
| Make changes to the client list to ensure a more even distribution of clients among staff |  |
| Reorganise work tasks to reduce emotional load for employees |  |
| Clarify employees’ roles and responsibilities |  |
| Facilitate work tasks to avoid heavy lifting and/or monotonous work |  |
| Increase employee participation through strengthening the work environment committee |  |
| Develop plans and routines to protect employees from threats and violence |  |
| Develop plans and routines to protect employees from unwanted sexual attention |  |
| Other, please specify: | |

**#6b Where any of the measures mentioned above implemented before attending the workshop?**

| Yes |  |
| --- | --- |
| No |  |
| Do not know |  |

**#6c If you are not implementing any measures to improve the work environment, what is the reason behind this?**

| We recently implemented measures |  |
| --- | --- |
| We do not see the need to implement measures to improve the work environment |  |
| We had to postpone any implementation due to other resource demanding projects |  |
| Other, please specify:  ……………………….………………………………… | |

**#7 Have you recently sought information on occupational safety and health management?** (Check all that apply)

| No |  |
| --- | --- |
| Yes, from the occupational health service |  |
| Yes, from the municipality |  |
| Yes, from the Labour Inspection Authority website |  |
| Yes, from the National Institute of Occupational Health website |  |
| Yes, from the “Idébanken” website |  |
| Other, please specify:  ……………………………………………… | |

**#8**

| **Overall, has the workshop contributed to…** | To a very small degree | To a small degree | Neither | To a large degree | To a very large degree |
| --- | --- | --- | --- | --- | --- |
| …increased awareness of the importance of conducting work environmental risk assessments? | 1 | 2 | 3 | 4 | 5 |
| … increased skills to improve your work environment? | 1 | 2 | 3 | 4 | 5 |
| …. enhanced knowledge of work environmental laws and regulations? | 1 | 2 | 3 | 4 | 5 |

# **Questionnaire on systematic occupational safety and health management**

**#1 Have you recently implemented, or are you in the near future planning to implement, measures to improve the working environment at your workplace?**

| Yes, we have recently implemented measures |  |
| --- | --- |
| Yes, we are in the process of implementing measures |  |
| Yes, we are planning to implement measures by 2019 |  |
| No |  |
| Do not know |  |

**#1a If yes, which measures have you implemented or are planning to implement?** (Check all that apply)

| Reinforce the work of safety representatives and/or the occupational health services |  |
| --- | --- |
| Identifying hazards and assessing the risks at your workplace |  |
| Develop plans for a systematic approach to occupational safety and health management |  |
| Conduct training/courses to enhance employee competencies |  |
| Make changes to the shift/work plan and/or the distribution of shifts |  |
| Make changes to the client list to ensure a more even distribution of clients among staff |  |
| Reorganise work tasks to reduce emotional load for employees |  |
| Clarify employees’ roles and responsibilities |  |
| Facilitate work tasks to avoid heavy lifting and/or monotonous work |  |
| Increase employee participation through strengthening the work environment committee |  |
| Develop plans and routines to protect employees from threats and violence |  |
| Develop plans and routines to protect employees from unwanted sexual attention |  |
| Other, please specify: | |

**#3 Have you recently sought information on occupational safety and health management?** (Check all that apply)

| No |  |
| --- | --- |
| Yes, from the occupational health service |  |
| Yes, from the municipality |  |
| Yes, from the Labour Inspection Authority website |  |
| Yes, from the National Institute of Occupational Health website |  |
| Yes, from the “Idébanken” website |  |
| Other, please specify:  ……………………………………………… | |
