# Supplementary material for: Do regulatory tools instigate measures to prevent work-related psychosocial and ergonomic risk factors? A process evaluation of a Labour inspection authority trial in the Norwegian home-care services
Source: BMC Res Notes. 2022 Nov 18;15:349. doi: 10.1186/s13104-022-06244-4 (PMC9673432; doi:10.1186/s13104-022-06244-4)
Supplement: Supplementary file 4 — Additional file 4: Table S1. Contravened regulations in the inspection group. [file 13104_2022_6244_MOESM4_ESM.docx]

| **Supplementary table - Contravened regulations in the inspection group** | |
| --- | --- |
|  | **Municipalities**  **(N=29)**  **n (%)** |
| Has the organisation elected a safety representative? | 0 (0) |
| Has the safety representative been given adequate training? | 6 (21) |
| Is the safety representative included in the organisation’s planning and implementation of measures that affect the work environment? | 5 (17) |
| Does the employer ensure that employees and their representatives can participate in the systematic occupational health and safety work? | 5 (17) |
| Has the organisation prepared a plan for how occupational health services can assist it? | 8 (28) |
| Do employees with leadership responsibilities have the necessary competencies to address health and safety considerations within their area of responsibility? | 2 (7) |
| Has the employer evaluated whether the work-scheduling is sound, prioritises safety concerns, and does not expose employees to potentially negative physical or mental consequences? | 6 (21) |
| Has the employer conducted a risk assessment and, based on this assessment, prepared plans and implemented necessary measures to prevent employees from being exposed to unhealthy work-related stress? | 12 (41) |
| Has the employer implemented routines for how non-conformities (undesirable events) are to be reported and followed up? | 3 (10) |
| Has the employer assessed the risk of employees working alone? | 16 (55) |
| Has the organisation implemented routines for how harassment or other improper conduct is to be prevented, reported, handled, and followed up? | 6 (21) |
| Has the employer conducted a risk assessment and, based on this, prepared a plan and implemented measures to protect employees from violence, threats of violence, or adverse social behaviour? | 27 (93) |
| Has the employer ensured that employees and their representatives have been adequately informed about risk factors related to violence and threats of violence, and implemented measures and routines for preventing, reporting, managing, and following up violence and threats of violence? | 11 (38) |
| Has the organisation developed protocols describing methods for handling violence, threats, or adverse social behaviour, including how such events should be prevented, reported, and managed? | 10 (34) |
| Have workers received appropriate training and practice so that they are protected, as far as possible, from violence and threats of violence? | 16 (55) |
| Has the employer conducted a risk assessment of factors in the working environment that may affect workers’ mental health? | 14 (48) |
| Has the employer implemented measures and/or prepared plans to reduce factors in the work environment that may affect workers’ mental health? | 13 (45) |
| Has the employer conducted a risk assessment of factors related to manual labour that can be harmful to employees’ health, including musculoskeletal complaints? | 11 (38) |
| Has the employer implemented measures and/or made plans to reduce or eliminate manual labour tasks that are taxing or harmful to employees’ health? | 10 (34) |
| Are employees who perform ergonomically taxing work, such as strenuous or repetitive tasks, given the necessary information and training? | 11 (38) |
